# Establishment of Outbreak Thresholds for Hepatitis A in South Africa Using Laboratory Surveillance, 2017–2020

**DOI:** 10.3390/v13122470

**Published:** 2021-12-10

**Authors:** Nishi Prabdial-Sing, Villyen Motaze, Jack Manamela, Kerrigan McCarthy, Melinda Suchard

**Affiliations:** 1 Division of the National Health Laboratory Service, National Institute for Communicable Diseases, Johannesburg 2131, South Africa; villyenm@nicd.ac.za (V.M.); jackm@nicd.ac.za (J.M.); kerriganm@nicd.ac.za (K.M.); melindas@nicd.ac.za (M.S.); 2 Faculty of Health Sciences, School of Pathology, University of Witwatersrand, Johannesburg 2000, South Africa; 3 Department of Global Health, Division of Epidemiology and Biostatistics, Faculty of Medicine and Health Sciences, Stellenbosch University, Cape Town 7935, South Africa

**Keywords:** hepatitis A, incidence, threshold, outbreak, surveillance

## Abstract

As South Africa transitions from endemic to intermediate endemicity, hepatitis A surveillance needs strengthening to monitor trends in disease incidence and to identify outbreaks. We used passive laboratory-based surveillance data from the National Health Laboratory Services to calculate national hepatitis A incidence and to establish thresholds for outbreaks. Incidence was calculated by age and geographic location. The static threshold used two or three standard deviations (SDs) above the mean hepatitis A incidence in 2017–2019, and a cumulative summation (CuSum2) threshold used three SDs above the mean of the preceding seven months. These thresholds were applied to hepatitis A data for 2020. From 2017 to 2020, the mean incidence of hepatitis A IgM was 4.06/100,000 and ranged from 4.23 to 4.85/100,000 per year. Hepatitis A incidence was highest in the Western Cape province (WCP) (7.00–10.92/100,000 per year). The highest incidence was in the 1–9-year-olds. The incidence of hepatitis A in 2020 exceeded the static threshold in two districts of the WCP: Cape Winelands in January and Overberg district in August. The provincial incidence did not exceed the static and CuSum2 thresholds. District-level analysis using either threshold was sensitive enough to monitor trends and to alert district health authorities, allowing early outbreak responses.

## 1. Introduction

Worldwide, 1.4 million people are affected with the hepatitis A virus (HAV) annually [1]. HAV causes acute disease of the liver, with clinical presentations ranging from asymptomatic to mild and occasionally severe. HAV is an enteric virus that is commonly spread through infected food and/or water. Hepatitis A prevalence is endemic in low- and middle-income countries, where clean sanitation and drinking water are lacking. Hepatitis A infection usually has a long incubation period of around 28 days (range 15–50 days) [2]. Serologic testing for hepatitis A immunoglobulin M (IgM) is required to confirm the diagnosis of an acute infection. Hepatitis A IgM can be detected 5–10 days before the onset of symptoms and can persist for up to 6 months [2].

The World Health Organization (WHO) has defined various levels of endemicity by immunity levels to HAV. A highly endemic area is one in which hepatitis A seroprevalence, measured by immunoglobulin G (IgG) or total antibody to HAV, is ≥90% by 10 years of age. An intermediate prevalence area is one in which seroprevalence is less than 90% by age 10 years, but reaches ≥50% by 15 years of age, while a low endemic area has seroprevalence under 50% by 15 years, but ≥50% by 30 years, and in a very low endemic area, seroprevalence remains under 50% by 30 years of age [3].

Most regions in Africa and Southeast Asia are highly endemic [4], with most infections occurring in children younger than 10 years of age, usually without symptoms. Following infection, children are usually protected with lifelong immunity, resulting in rare outbreaks amongst adults [5]. In high-income countries, such as England and USA, seroprevalence rates are low [4], and the age of first infection is later and often symptomatic. Where adults are non-immune, outbreaks occur in high-risk groups, such as injecting drug users, men who have sex with men, and homeless people [6,7]. South Africa is transitioning to intermediate endemicity, whereby IgG seroprevalence is ≥50% by 15 years, but less than 90% by age 10 years [8].

The WHO recommends that in countries that are transitioning from high to intermediate endemicity, large-scale vaccination programs may be cost-effective and beneficial [3]. Currently, in South Africa, the hepatitis A vaccine is only provided in the private health sector and is not part of the national expanded program on immunization (EPI). In the private sector, estimated at less than 15% of the population, the hepatitis A vaccine is given at 12 and 18 months of age [9].

Hepatitis A infection is a notifiable medical condition (NMC) in South Africa, requiring notification by clinicians and by testing laboratories. This includes the National Health Laboratory Service (NHLS), which serves approximately 80% of persons resident in South Africa. National surveillance of hepatitis A infection in South Africa is important to monitor age trends in disease incidence, and to identify outbreaks so as to intervene and halt chains of transmission. However, until recent strengthening of the notifiable medical diseases system, hepatitis A outbreak reports have depended on case-based surveillance and local care providers who identify epidemiological linkages between cases, with no systematic monitoring of geographic trends in incidence. This means that opportunities to identify clusters of cases, and thus, prevention of outbreaks through early diagnosis, implementation of infection prevention and control, and provision of post-exposure prophylaxis are lost. As an example, a local outbreak of acute hepatitis A infection was actively notified to the National Institute for Communicable Disease (NICD) in 2020. In this outbreak, 22 cases of acute hepatitis A infection were recorded in the Theewaterskloof sub district, Overberg district, Western Cape province from 1 July to 17 August 2020. Age ranged from 2 to 61 years, with a median of 20 (IQR 15–34 years). The age group 15–19 years comprised the highest frequency (*n* = 5.23%), with males and females in almost equal proportion (10 males and 12 females), and two fatalities, aged 14 and 57. There were burst water pipes and blocked drains reported in the area where the cases were investigated [10].

Several statistical methods have been previously used to detect outbreaks using summary statistics, such as means and standard deviations, or cumulative summations (CuSum, [11]). Summary statistics can be used to review historical data, using the mean and standard deviation (SD) [12,13] to form a baseline over a period of weeks, months, or years.

The CuSum method uses data over a rolling period to inform of an expected or predicted level [14], and was utilized in the Early Aberration Reporting System of the Centers for Disease Control and Prevention, US [12,15,16]. CuSum C1, C2, and C3 algorithms indicate the degree of sensitivity of the calculation [15], with C1 being the least sensitive (using seven or more data points, including all data points) and C3 being the most sensitive (with seven data points, including a mean of the three most recent data points). Algorithms C2 and C3 provide buffer zones as the last two data points are removed from the analyses, as they may show slow increases in incidence that may alter the threshold [12]. The CuSum method is considered easy to calculate and to automate, and can be used with either large or small datasets [11]. Other approaches include a historical limits method [12] and the Salmonella Potential Outbreak Targeting [17] method. Similar methods of moving thresholds were applied to influenza, using a moving epidemic method (MEM, [18]) and a moving percentile method for hand, foot, and mouth diseases, mumps, influenza, scarlet fever, chickenpox, and rubella [19].

In this study, we utilized the number of laboratory-confirmed hepatitis A cases nationally from 2017 to 2019 to calculate testing and incidence rates, and to determine thresholds for public health action. We tested the sensitivity of these thresholds to detect the above-mentioned hepatitis A outbreak reported from the Overberg district in the Western Cape province.

## 2. Methods

We performed passive laboratory-based surveillance using the NHLS Corporate Data Warehouse (CDW). NHLS CDW is a centralized system from which data on all laboratory tests performed at NHLS Laboratories throughout South Africa can be accessed (ethical approval from the Human Research Ethics Committee, HREC University of Witwatersrand, 160667). We analyzed data from 1 January 2017 to 31 December 2020 after removal of duplicates. Deduplication was performed by removing data with the same identifiers, such as hospital numbers, name and surname, date of birth, and age. Data from private laboratories were not included in the analysis. Data from three districts (Alfred Nzo in the Eastern Cape, Xhariep in the Free State, and Francis Baard in the Northern Cape) were missing in the 2018 dataset and were excluded from the analyses. During the years of data collection (2017–2019), the uThungulu District Municipality was renamed the King Cetshwayo District Municipality in the KwaZulu Natal province. Entries missing data on age, province, and district variables were also excluded from the analyses.

We used mid-year national population statistics [20,21,22,23] to calculate the testing rate per 100,000. The positivity rate was calculated by dividing the number of hepatitis A IgM positive cases by the total number of hepatitis A IgM tests done during the same year and reported as a percentage. Similarly, the incidence rate per 100,000 was calculated as the number of hepatitis A IgM positive tests/100,000 population per year. We also calculated provincial and age-adjusted incidence rates (Microsoft Excel 2016, Microsoft Corporation, Washington, DC, USA).

The first three years (2017–2019) of historical data were used to calculate a baseline (and we refer to this as a static threshold). Two standard deviations above the mean (mean + 2SD) [12] and three standard deviations (mean + 3SD) of the historical data [13] were used as our measures of an alert level and an action level at provincial and district levels, respectively. We compared these to the CuSum2 threshold, which displays a medium threshold that used the mean + 3SD for each rolling 7-month period (excluding the last two most recent points) as an action level (Microsoft Excel 2016, Microsoft Corporation, Washington, DC, USA).

We interrogated data from 2020 to determine whether provincial or district incidence exceeded the established thresholds using the Overberg district outbreak in August 2020 as a case study.

## 3. Results

### 3.1. Hepatitis A Positivity, Incidence, and Testing Rates

During the period 2017–2020, we identified 493,212 specimens submitted to NHLS laboratories for hepatitis A IgM testing, of which 13,914 were duplicates or had missing data, and were deleted from the analyses. A total of 479,298 records were analyzed.

The testing rate fluctuated from 151.46 (95%CI, 150.46–152.47) to 254.03 (95%CI, 252.73–255.35) tests/100,000/year (Table 1). Over the years, the positivity rate ranged from 0.83% in the North West province to 8.41% in the Western Cape province. The national incidence rate of confirmed hepatitis A cases ranged from 4.23 to 4.85/100,000 per year, with the exception of 2018, when the rate was 2.90/100,000. This apparently low incidence was due to missing data from the three districts in three provinces for 2018. The positivity rate was highest in 2020 (Table 1). The provincial incidence rate/100,000 population was highest in the Western Cape (ranging from 7.00 to 10.92), and the Western Cape province had a relatively low testing rate/100,000 over the years, compared to the other provinces. Limpopo, Mpumalanga, and Northern Cape provinces showed an increase in the incidence rate/100,000 over the years, while North West and Western Cape showed a decrease (Table 1).

### 3.2. Hepatitis A Incidence by Age Distribution and by Province

**Figure 1:**
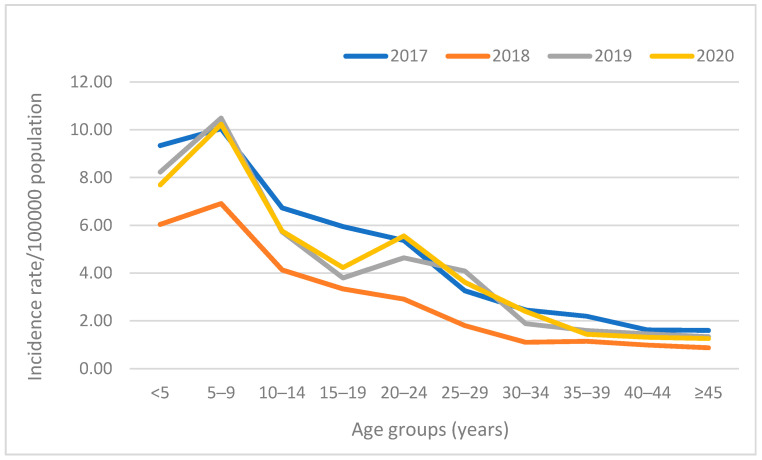
National age distribution of laboratory-confirmed hepatitis A cases per 100,000 population, 2017–2020.

There were cases amongst all age groups, but the age-stratified incidence rate was highest in the younger age groups (<5 years and 5–9 years). A peak in incidence was noted in the 20–24-year-old age group in 2020, in which the rate per 100,000 was higher than in 15–19-year-olds (Figure 1).

**Figure 2:**
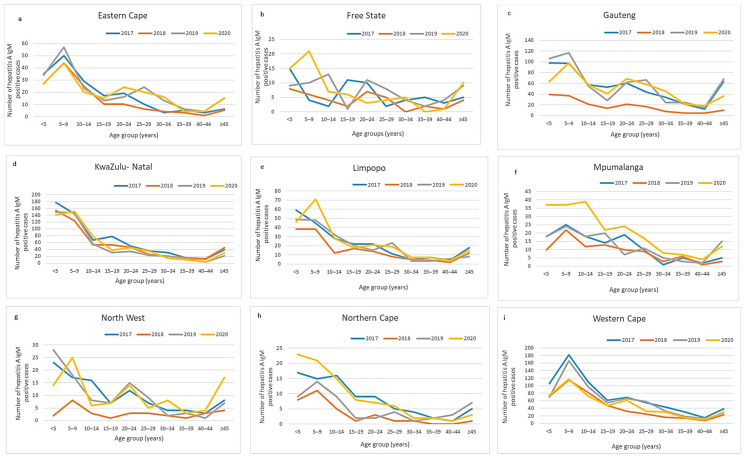
(**a**–**i**) Provincial age distribution of laboratory-confirmed hepatitis A cases, 2017–2020.

Provincial age-stratified case numbers showed similar trends over the years, with the highest number of cases in younger age groups (Figure 2a–i) and more cases in 5–9-year-olds than 1–5-year-olds. Western Cape and KwaZulu-Natal showed the highest absolute number of positive cases (Figure 2d,i). Eastern Cape, Gauteng, and North West provinces showed a trend to bimodal distributions, with a higher number of cases aged 20–24 years than 15–19 years in 2019–2020 (Figure 2a,c,g). Comparison of under-5′s and the 5–9-year-old groups amongst other provinces showed mixed patterns, depending on the year.

KwaZulu-Natal, Western Cape, and Gauteng provinces showed the highest case proportions of under 5′s and 5–9-year-olds compared with other age groups for all years (Figure 2d,i). At age groups above 40 years, Gauteng showed notably higher case numbers than the other provinces in 2017, 2019, and 2020 (Figure 2d).

### 3.3. Provincial Hepatitis A Incidence Thresholds

**Figure 3:**
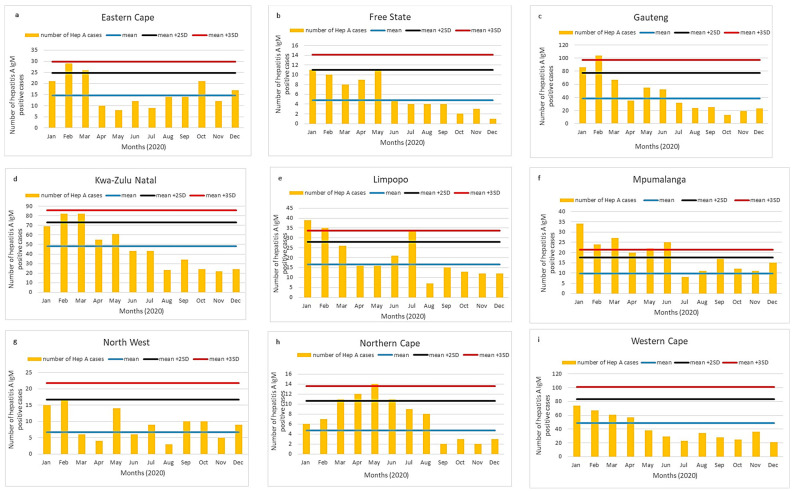
(**a**–**i**) Acute hepatitis A cases by province in South Africa, 2020. Number of acute hepatitis A cases in 2020 (orange bars) shown in comparison with mean and two (black line) or three (red line) standard deviations of data from 2017 to 2019.

**Figure 4:**
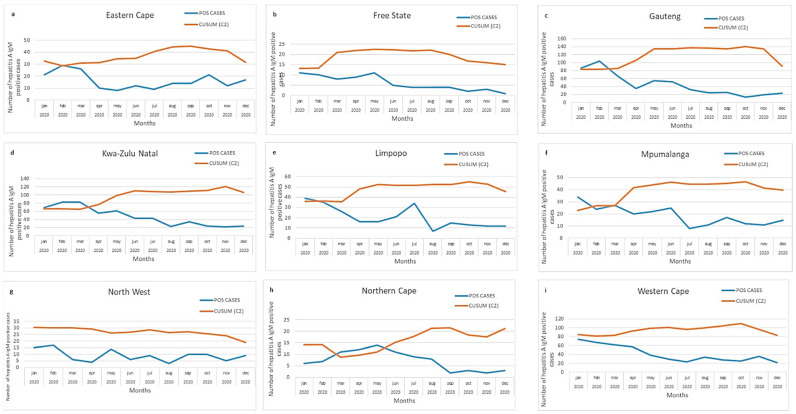
(**a**–**i**) Acute hepatitis A cases by province in South Africa, 2020. Number of hepatitis A cases (blue line) in comparison with threshold (orange line), calculated using CuSum2 method.

For each of the nine provinces in South Africa, we depicted a static threshold (Figure 3a–i) and CuSum2 thresholds (Figure 4a–i).

The number of hepatitis A IgM cases was higher in January to March 2020 compared to the rest of the year. This pattern was likely due to the impact on health-seeking behavior and population mixing as a result of the COVID-19 pandemic and the national lockdown, which began on 26 March 2021. Gauteng, Limpopo, Mpumalanga, and Northern Cape exceeded the mean + 3SD threshold in the early months of the year (Figure 3c,e,f,h). Mpumalanga showed cases exceeding the mean + 2SD consecutively for almost six months of 2020 (Figure 3f). Eastern Cape and KwaZulu-Natal showed numbers exceeding the mean + 2SD in February to March 2020 (Figure 3a,d).

Using the CuSum2 method, Gauteng, Mpumalanga, and KwaZulu Natal (Figure 4c,d,f) exceeded the threshold in the early months, while Northern Cape (Figure 4h) exceeded the threshold in March–May 2020. The CuSum2 method did not detect elevated cases in Limpopo or Eastern Cape (Figure 4a,e).

### 3.4. District Hepatitis A Incidence Thresholds

**Figure 5:**
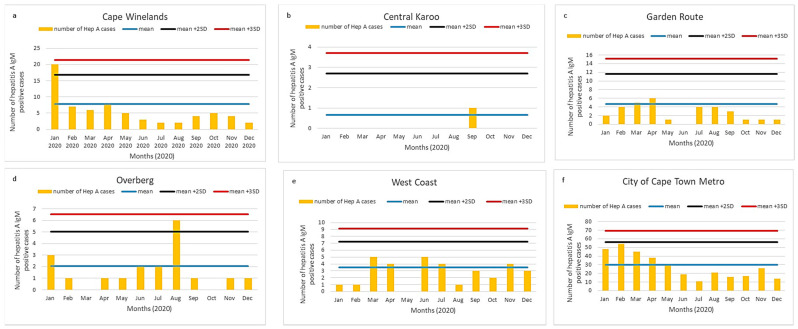
(**a**–**f**) Number of acute hepatitis A cases per district, Western Cape province, 2020. Number of acute hepatitis A cases (orange bars) are shown in comparison with the mean and two (black line) or three (red line) standard deviations of 2017–2019 data. A peak was noted in the Overberg district in August.

**Figure 6:**
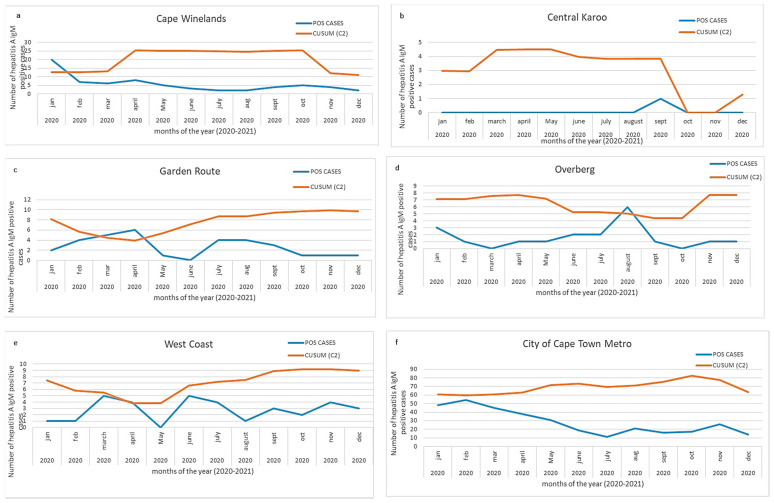
(**a**–**f**) Acute hepatitis A cases per district, Western Cape province, 2020. Number of hepatitis A cases (blue line) in comparison with threshold (orange line) calculated using CuSum2 method. A peak was noted in Overberg district in August and Garden Route district in April.

The Western Cape province had the highest incidence per 100,000 population for each year from 2017 to 2020 (Table 1); we assessed district-level case numbers for each of six districts in the Western Cape. The number of cases for each district is shown in Figure 5a–e and Figure 6a–e in comparison with district data from three prior years.

Of the six districts in the Western Cape province, two districts showed an increase in hepatitis A IgM positive cases, exceeding the mean + 2SD threshold: Cape Winelands in January 2020 (Figure 5a) and the Overberg district in August 2020 (Figure 5d). Both peaks were also detected using the CuSum2 method (Figure 6a,d). The CuSum2 threshold also flagged the Garden Route in March 2020 as another district with cases exceeding the threshold (Figure 6c), despite case numbers in the Garden Route being below the mean of the prior 3-year period (Figure 5c).

For the other eight provinces, district thresholds are shown in Supplementary Figure S1A–H. Whilst most districts showed an increase in cases at the beginning of 2020 (Supplementary Figure S1A,C,E,G), certain districts showed a surge in mid-year (the Umkhanyakude district in KwaZulu-Natal, Supplementary Figure S1D and the Pixley Ka Seme district in Northern Cape, Supplementary Figure S1H), which was also seen on CuSum2 plots (data not shown). The surge in cases in Mpumalanga (Figure 3f) was seen in all three districts, particularly Ehlanzeni and Gert Sibande (Supplementary Figure S1F).

## 4. Discussion

We utilized the number of laboratory-confirmed hepatitis A IgM positive cases nationally from 2017 to 2020 to calculate testing and incidence rates, and used 2017–2019 data to determine thresholds for public health action. We tested the sensitivity of these thresholds to detect the above-mentioned hepatitis A outbreak reported from the Overberg district in the Western Cape province for 2020.

We reported a relatively stable national incidence and age-related rate of hepatitis A infection from 2017 to 2020 of 4 cases/100,000 and 6–10/100,000 in the 1–9-year age group. The highest provincial incidence was consistently observed from the Western Cape province (7–10 cases/100,000), whilst increases in incidence over the years were observed in Limpopo, Mpumalanga, and the Northern Cape provinces, with the Northern Cape almost tripling its incidence rate from 2017 to 2020. The static and CuSum2 thresholds successfully identified the outbreak in the Overberg district. Throughout the study period, the highest number of positive cases per province was amongst the under-5 and 5–9 age groups, although an increasing number of cases was seen in the older age groups, particularly in Western Cape and Gauteng provinces.

The access to safer water and sanitation, and better living standards in South Africa are possibly reducing the high endemicity at a country level to intermediate levels, thereby accounting for the adult cases reflected in our data. Paradoxically, unequal access to safe water, sanitation, and adequate hygiene is still seen in many rural communities and informal settlements in urban areas of South Africa [24]. Three percent of South African households still used water from rivers, stagnant water sources, dams, and wells in 2018–2019 [25]. In 2016, the City of Cape Town had placed around 370 chemical toilets to accommodate the approximately 60,000 people living in the Marikana informal settlement, equating to one toilet for every 32 households [26]. Many service delivery challenges still face South Africans, which include service provision by struggling municipalities, lack of maintenance and infrastructure, as well as backlogs, as the population increases and informal settlements expand [27], and these may drive the outbreaks at district and provincial levels, especially in older age groups.

Like South Africa, many other middle-income countries such as China, Brazil, and India have transitioned from high endemic to intermediate levels of hepatitis A seroprevalence [28,29,30]. The transition from very high to high endemicity was shown as early as 2014 in a report from eighteen studies in 10 countries in West Africa (1970–2013), with a shift in hepatitis A prevalence rates from younger children (<5 years old) to older children (5–14 years old) in countries with high gross domestic products (GDPs) [31]. Similar recent findings were reported in a meta-analysis on 32 studies in 13 countries across Africa [32] from 2008 to 2019, which indicated that immunity transitions from young to adolescent age groups may change Africa’s overall high endemicity of hepatitis A. Many of the studies used in these systematic reviews were cohort or cross-sectional on sample sizes varying from 9 to 3000, with only one study in South Africa, which looked at a national population [8]. With the improvement of socio-economic status and the deliverance on the sustainable developmental goals on improved sanitation and clean water for all [5], a reliable source of national data is needed in many African countries to monitor shifts in age-specific immunity; also needed are set local national thresholds for the rapid detection of outbreaks.

We applied two different approaches to establish alert thresholds based on an increase in the number of cases at the provincial or district levels. The static threshold used historical annual data over three years (2017–2019) to identify an alert level (mean + 2SD) and an action level (mean + 3SD) [15]. These thresholds were used to check if the number of positive cases for the year 2020 reached alert levels. The CuSum2 method used data from the previous seven months to set a threshold for current data and, in this way, reflected a rolling change in the set threshold [14,15].

At the provincial level, we showed that both static and CuSum2 thresholds showed alert or action levels for the Gauteng and Northern Cape provinces. With the static thresholds, we found cases in 2020 exceeding action levels (mean + 3SD) for Eastern Cape, Limpopo, and Mpumalanga. In comparison, the surges in the above-mentioned provinces did not exceed the CuSum2 threshold. The likely reason for this is the sustained higher number of cases in the previous months, which adjusted the CuSum2 threshold. Thus, the mean + 3SD method appeared to be more sensitive in detecting outbreaks at the provincial level. However, at the provincial level, neither method was sensitive enough to identify the localized cluster in the Overberg, Western Cape region.

At the district level, the Overberg cluster was clearly detected by both the mean + 3SD and CuSum2 methods. Of the six districts plotted for the Western Cape province, two districts, Cape Winelands and Overberg, showed action levels using both of the methods. An additional surge of cases was visible using the CuSum2 threshold for the Garden Route district; however, such cases were just above the mean number of cases in the Garden Route district for the prior three years and, therefore, can be considered a false positive detection. We, therefore, recommend the use of the simple mean + 2 or 3SD method, as it was equally sensitive at the provincial and district levels, but resulted in fewer false action alerts than the CuSum2 method. We also plotted monthly mean + 2 or 3SDs over 2017–2019 to see whether seasonal influences required a baseline, which shifted depending on the month of the year (data not shown); however, the shifting baseline added complexity to the interpretation of alert or action levels and was not intuitive to interpret. We, therefore, plan to utilize the annual baseline of + 2 or 3SDs as our alert and action thresholds for future outbreak detection.

Limitations of this work include the fact that the data do not include results from medically insured persons who accessed private healthcare. The data mining process is estimated as ~80–90% accurate at assigning duplicates, completeness, and consistency [33], and deficiencies were seen in our 2018 data. One advantage of using a static annual threshold based on historical data is its relative simplicity and easy interpretation. Variations in surveillance methods and changing disease epidemiology over time can, however, limit the use of an unchanging baseline [14] and will need a review after every 4–5 years. If historical data were not available, then CuSum would be advantageous to identify outbreaks, using only recent data.

## 5. Conclusions

Endemicity of hepatitis A in South Africa varies by region of the country. As South Africa transitions to intermediate endemicity, more frequent hepatitis A outbreaks should be anticipated, as the average age of infection shifts from children to older age groups in which disease is more severe. We have shown two methods of using national laboratory-based hepatitis A incidence-surveillance data to monitor trends and to alert district health authorities, allowing early outbreak responses. Hepatitis A incidence data will also be essential for decisions regarding the need for hepatitis A vaccine introduction into the immunization schedule.

## Figures and Tables

**Table 1:** National Hepatitis A positivity rate, incidence, and testing rate for years 2017 to 2020.

**2017**						
**Province in South Africa**	**Population (Mid-Year)**	**Hep A IgM Tests**	**Hep A IgM Pos**	**Positivity Rate (%)**	**Testing Rate/100,000**	**Incidence Rate/100,000**
Eastern Cape	6,498,700	15,247	178	1.17	234.62	2.74
Free State	2,866,700	4333	61	1.41	151.15	2.13
Gauteng	14,278,700	40,653	583	1.43	284.71	4.08
KwaZulu-Natal	11,074,800	38,721	665	1.72	349.63	6.00
Limpopo	5,778,400	10,112	226	2.23	175.00	3.91
Mpumalanga	4,444,200	13,664	128	0.94	307.46	2.88
North West	1,214,000	6398	105	1.64	527.02	8.65
Northern Cape	3,856,200	4090	84	2.05	106.06	2.18
Western Cape	6,510,300	10,368	711	6.86	159.26	10.92
**Total**	**56,521,900**	**143,586**	**2741**	**1.91**	**254.04**	**4.85**
**2018**						
Eastern Cape	6,522,700	8871	136	1.53	136.00	2.09
Free State	2,954,300	2090	40	1.91	70.74	1.35
Gauteng	14,717,000	14,079	188	1.34	95.66	1.28
KwaZulu-Natal	11,384,700	37,827	564	1.49	332.26	4.95
Limpopo	5,797,300	6629	156	2.35	114.35	2.69
Mpumalanga	4,523,900	7717	92	1.19	170.58	2.03
North West	3,979,000	3625	30	0.83	91.10	0.75
Northern Cape	1,225,600	1432	32	2.23	116.84	2.61
Western Cape	6,621,100	5163	434	8.41	77.98	6.55
**Total**	**57,725,600**	**87,433**	**1672**	**1.91**	**151.46**	**2.90**
**2019**						
Eastern Cape	6,712,276	12,350	211	1.71	183.99	3.14
Free State	2,887,465	4618	71	1.54	159.93	2.46
Gauteng	15,176,115	37,552	615	1.64	247.44	4.05
KwaZulu-Natal	11,289,086	33,792	509	1.51	299.33	4.51
Limpopo	5,982,584	10,149	213	2.10	169.64	3.56
Mpumalanga	4,592,187	9544	134	1.40	207.83	2.92
North West	4,027,160	7379	104	1.41	183.23	2.58
Northern Cape	1,263,875	3769	54	1.43	298.21	4.27
Western Cape	6,844,272	10,453	602	5.76	152.73	8.80
**Total**	**58,775,020**	**129,606**	**2513**	**1.94**	**220.51**	**4.28**
**2020**						
Eastern Cape	6,734,001	11,377	193	1.70	168.95	2.87
Free State	2,928,903	4409	72	1.63	150.53	2.46
Gauteng	15,488,137	32,847	535	1.63	212.08	3.45
KwaZulu-Natal	11,531,628	30628	562	1.83	265.60	4.87
Limpopo	5,852,553	9830	246	2.50	167.96	4.20
Mpumalanga	4,679,786	10,361	226	2.18	221.40	4.83
North West	4,108,816	6155	108	1.75	149.80	2.63
Northern Cape	1,292,786	3340	88	2.63	258.36	6.81
Western Cape	7,005,741	9726	493	5.07	138.83	7.04
**Total**	**59,622,351**	**118,673**	**2523**	**2.13**	**199.04**	**4.23**

## Data Availability

The data presented in this study are available on request from the corresponding author. The data are not publicly available due to the data being the property of the NHLS.

## Supplementary Materials

The following are available online at https://www.mdpi.com/article/10.3390/v13122470/s1, Supplementary Figure S1A–H. Acute hepatitis A cases by district for each of the other eight provinces in South Africa, 2020. Number of acute hepatitis A cases in 2020 (orange bars) shown in comparison with mean and two (black line) or three (red line) standard deviations of data from 2017–2019. Eastern Cape (S1A), Free State (S1B), Gauteng (S1C), KwaZulu Natal (S1D), Limpopo (S1E), Mpumalanga (S1F), North West (S1G) and Northern Cape (S1H).
