# Supplementary material for: Establishment of Outbreak Thresholds for Hepatitis A in South Africa Using Laboratory Surveillance, 2017–2020
Source: Viruses. 2021 Dec 10;13(12):2470. doi: 10.3390/v13122470 (PMC8704411; doi:10.3390/v13122470)
Supplement: Supplementary file 1 [file viruses-13-02470-s001.zip › viruses-1454985-supplementary.pdf]

Figure S1A. Eastern Cape Province

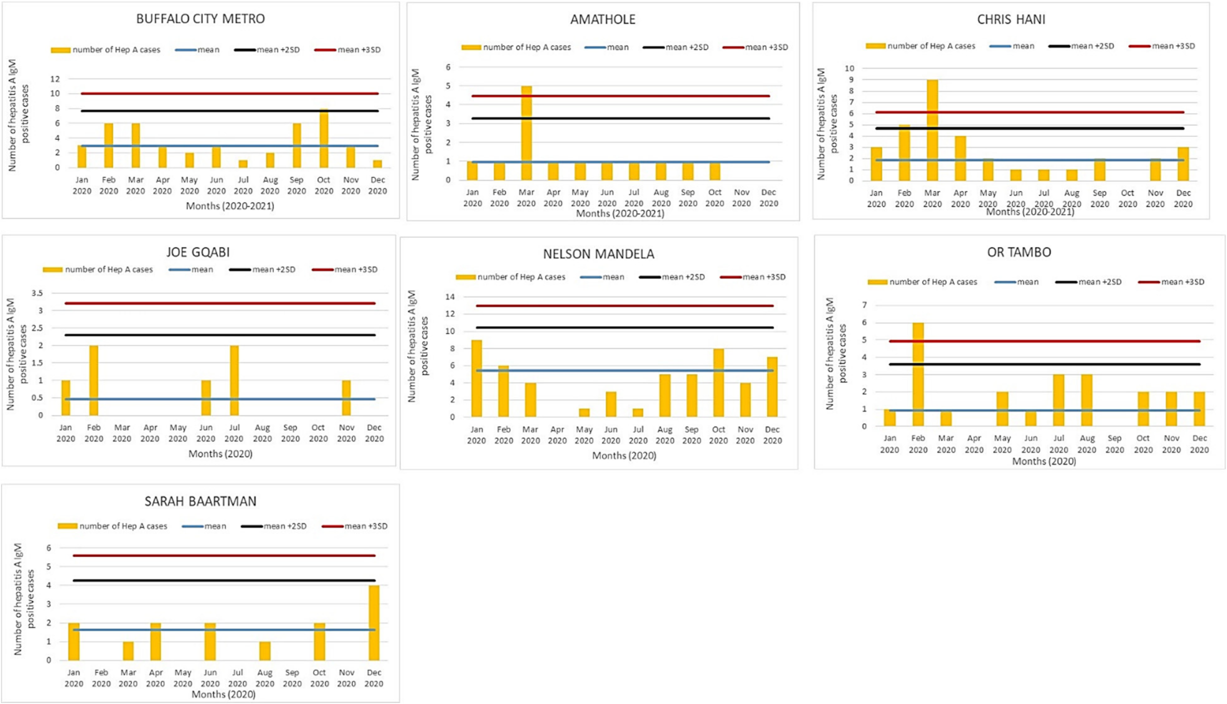

Data for 2018 from Alfred Nzo district was missing

Figure S1B. Free State Province

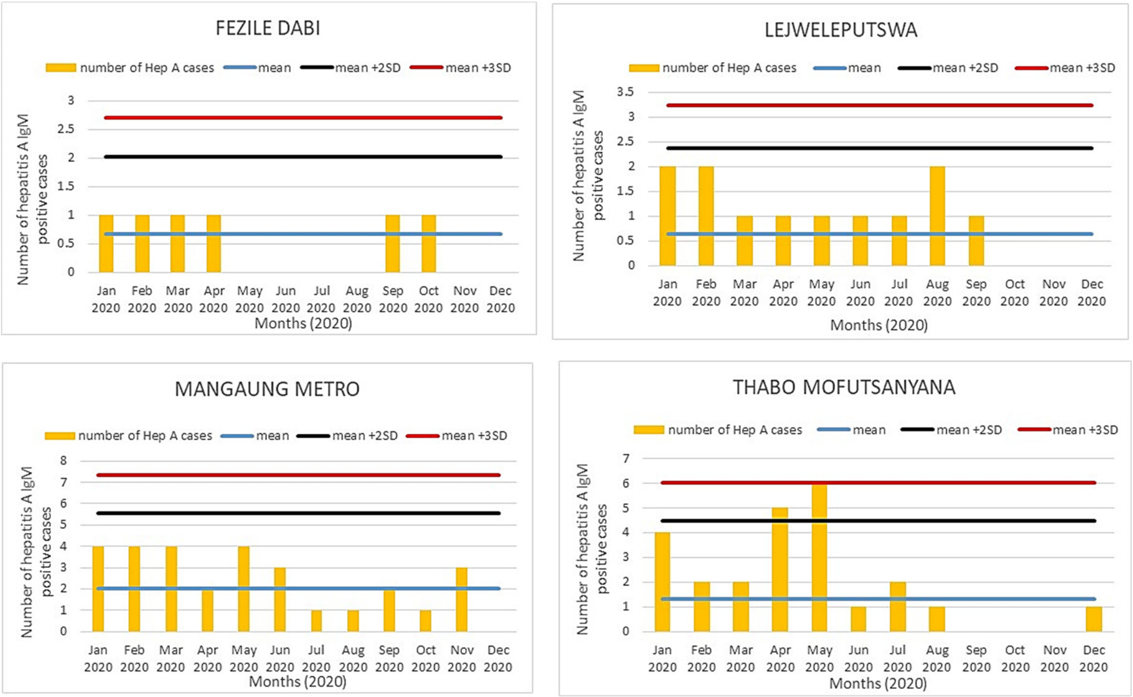

Data for 2018 from Xhariep district was missing

Figure S1C. Gauteng Province

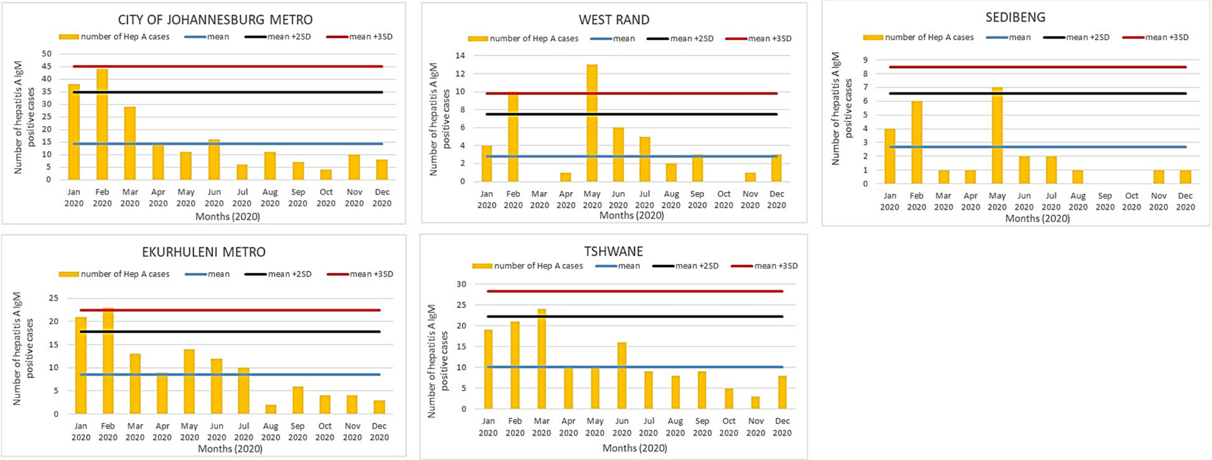

Figure S1D. KwaZulu –Natal Province

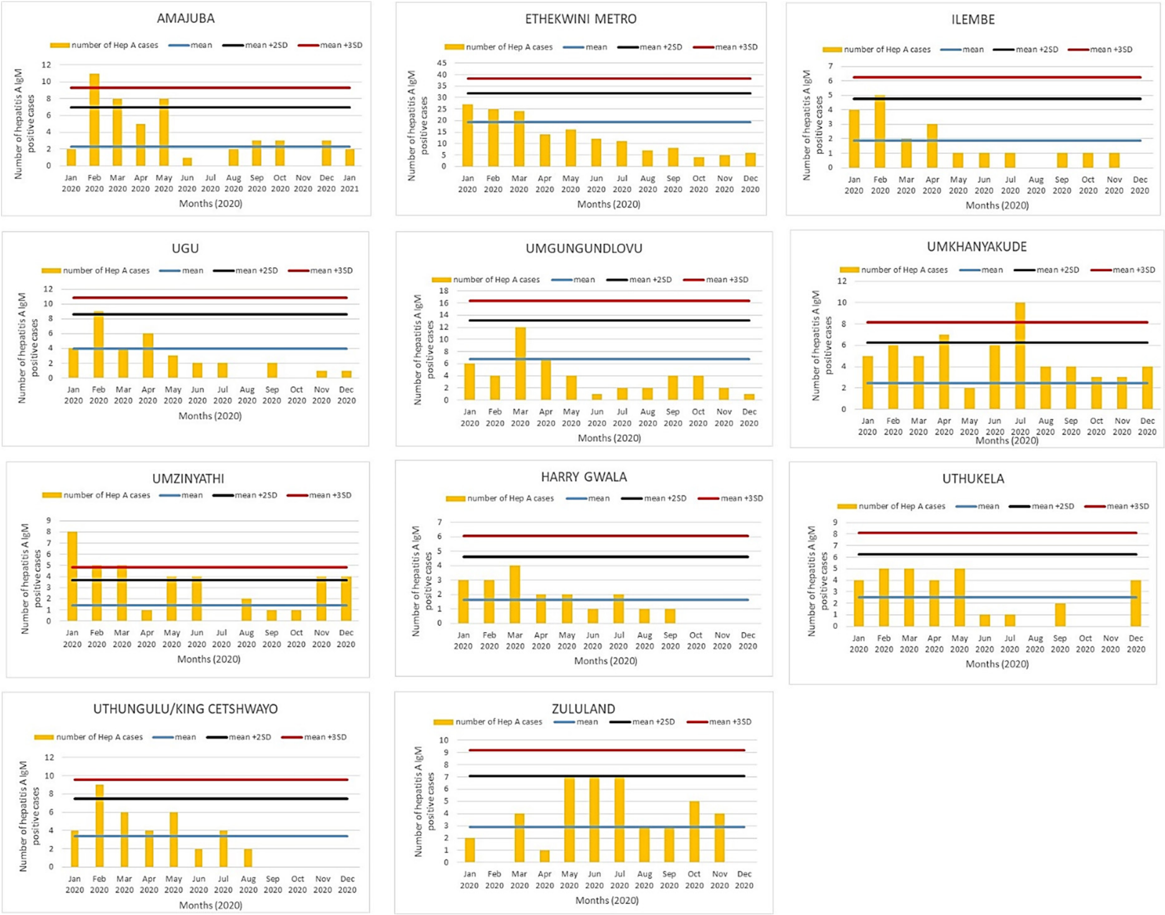

Figure S1E. Limpopo Province

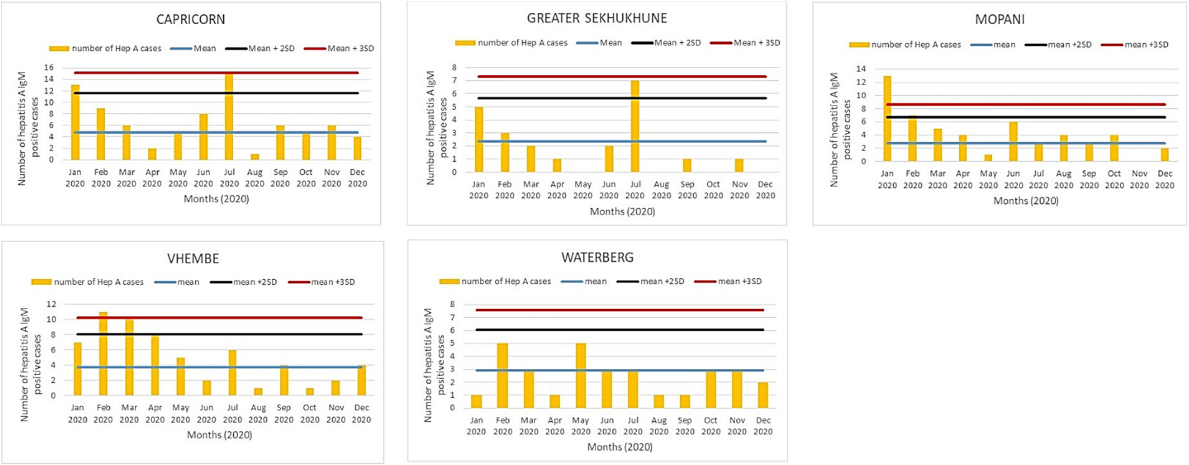

Figure S1F. Mpumalanga Province

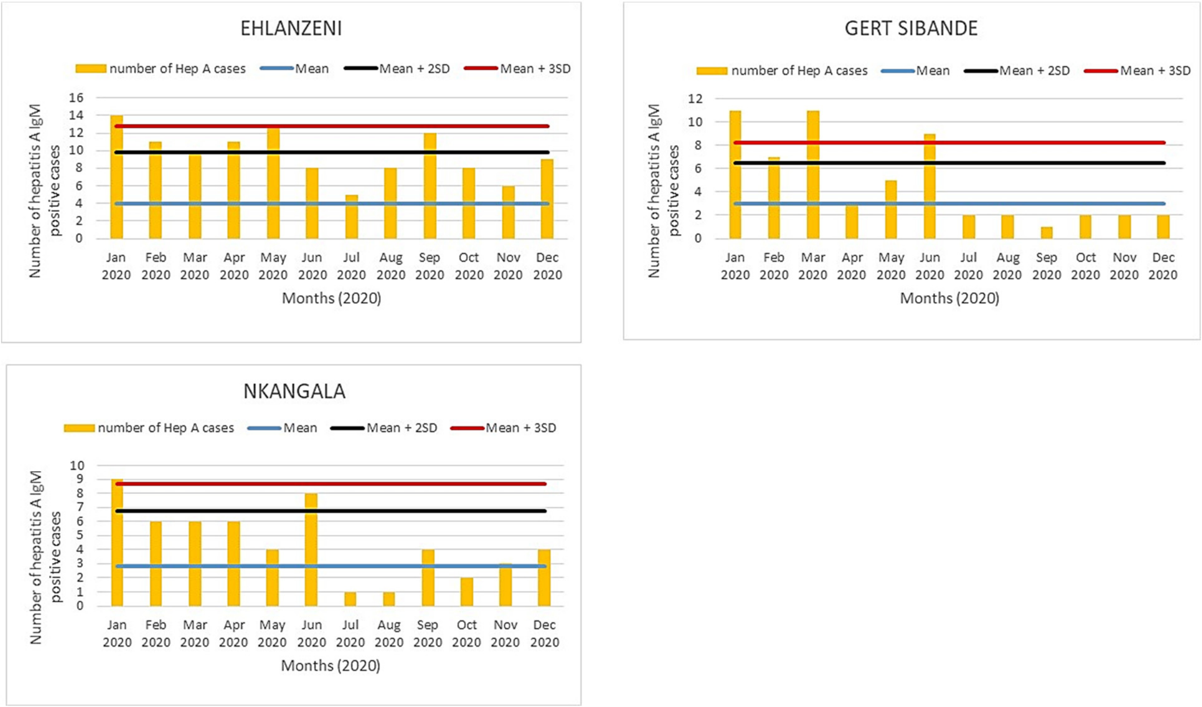

Figure S1G. North West Province

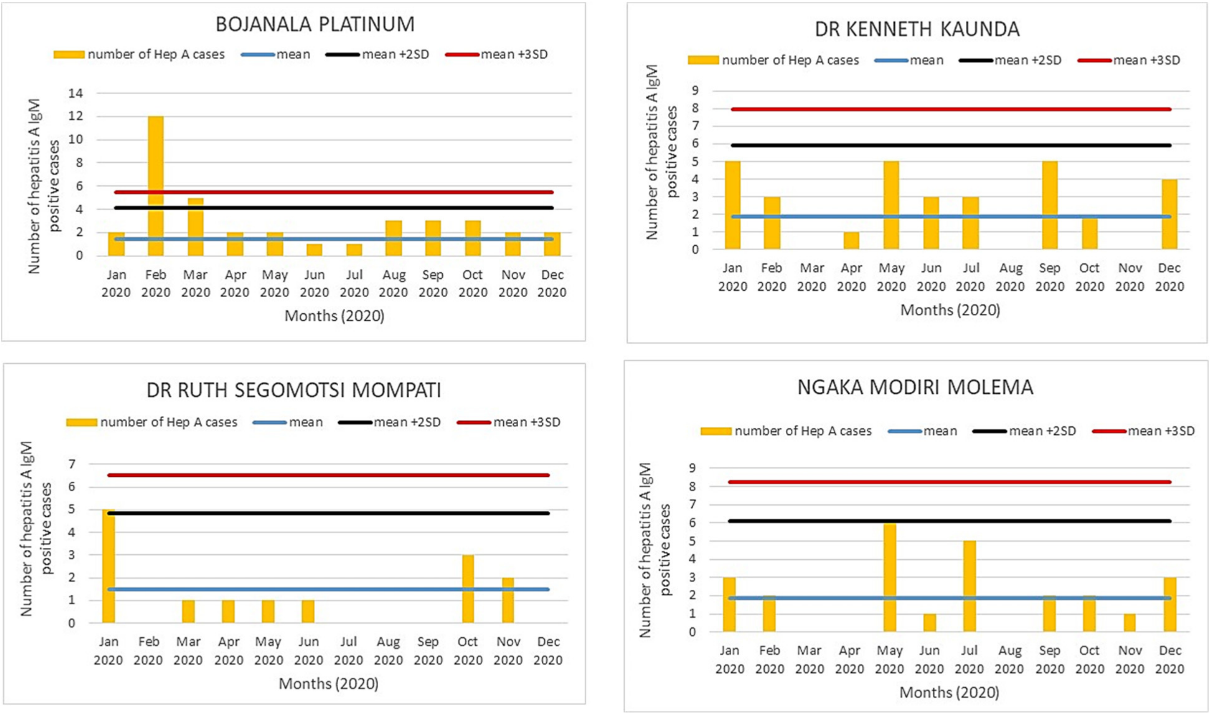

Figure S1H. Northern Cape Province

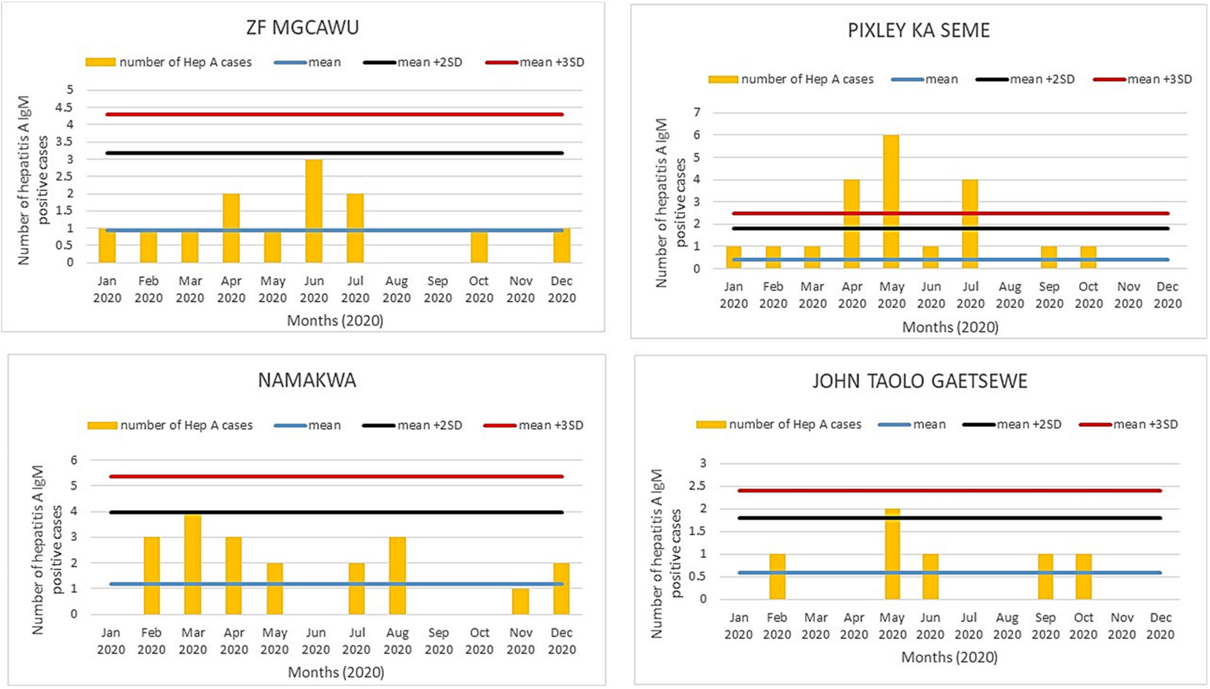

Data for 2018 from Frances Baard was missing

Supplementary Figures S1A–H. Acute hepatitis A cases by district for each of the other eight provinces in South Africa, 2020. Number of acute hepatitis A cases in 2020 (orange bars) shown in comparison with mean and two (black line) or three (red line) standard deviations of data from 2017-2019. Eastern Cape (S1A), Free State (S1B), Gauteng (S1C), KwaZulu Natal (S1D), Limpopo (S1E), Mpumalanga (S1F), North West (S1G) and Northern Cape (S1H).
